# Convolutional neural network for high-accuracy functional near-infrared spectroscopy in a brain–computer interface: three-class classification of rest, right-, and left-hand motor execution

**DOI:** 10.1117/1.NPh.5.1.011008

**Published:** 2017-09-14

**Authors:** Thanawin Trakoolwilaiwan, Bahareh Behboodi, Jaeseok Lee, Kyungsoo Kim, Ji-Woong Choi

**Affiliations:** Daegu Gyeongbuk Institute of Science and Technology, Department of Information and Communication Engineering, Daegu, Republic of Korea

**Keywords:** functional near-infrared spectroscopy, brain–computer interface, support vector machine, artificial neural network, convolutional neural network, feature extraction

## Abstract

The aim of this work is to develop an effective brain–computer interface (BCI) method based on functional near-infrared spectroscopy (fNIRS). In order to improve the performance of the BCI system in terms of accuracy, the ability to discriminate features from input signals and proper classification are desired. Previous studies have mainly extracted features from the signal manually, but proper features need to be selected carefully. To avoid performance degradation caused by manual feature selection, we applied convolutional neural networks (CNNs) as the automatic feature extractor and classifier for fNIRS-based BCI. In this study, the hemodynamic responses evoked by performing rest, right-, and left-hand motor execution tasks were measured on eight healthy subjects to compare performances. Our CNN-based method provided improvements in classification accuracy over conventional methods employing the most commonly used features of mean, peak, slope, variance, kurtosis, and skewness, classified by support vector machine (SVM) and artificial neural network (ANN). Specifically, up to 6.49% and 3.33% improvement in classification accuracy was achieved by CNN compared with SVM and ANN, respectively.

## 1 Introduction

### 1.1 Brain–Computer Interface

A brain–computer interface (BCI) is a means of communication between the human brain and external devices. BCIs are typically designed to translate the neuronal activity of the brain to restore motor function, or to control devices.^1^^–^^9^ The major components of an effective BCI system are: (1) acquisition of brain signals using a neuroimaging modality, (2) signal processing and analysis to obtain features representative of the signal, and (3) translation of features into commands to control devices.^7^ Well-designed BCI systems have proven to be helpful for patients with severe motor impairment, and have improved their quality of life. For instance, many studies have been successfully conducted with patients who have suffered a stroke,^10^^,^^11^ have amyotrophic lateral sclerosis,^12^^,^^13^ or have spinal cord injury (SCI),^14^^,^^15^ in which BCI systems have allowed them to control external devices.

BCI systems have been developed based on invasive^16^^,^^17^ as well as noninvasive^4^^,^^18^ neuroimaging modalities, including electroencephalography (EEG),^18^^–^^23^ magnetoencephalography (MEG),^10^^,^^24^ electrocorticography (ECoG),^16^ functional magnetic resonance imaging (fMRI),^25^^–^^27^ and functional near-infrared spectroscopy (fNIRS).^12^^,^^23^^,^^28^^–^^33^ BCI systems based on MEG, ECoG, fMRI, and EEG generally suffer from bulkiness, high cost, high sensitivity to head movements, low spatial and temporal resolution, and low signal quality. fNIRS-based systems are known to be more advantageous, in that they can provide moderate temporal and spatial resolution.

### 1.2 Functional Near-Infrared Spectroscopy-Based Brain–Computer Interface System

In the last few decades, fNIRS has been recognized as a promising noninvasive optical imaging technique for monitoring the hemodynamic response of the brain using neurovascular coupling. Neurovascular coupling in the cerebral cortex captures the increases in oxygenated hemoglobin (HbO) and reductions in deoxygenated hemoglobin (HbR) that occur during brain activity. To accomplish this, fNIRS employs multiple light sources and detectors, which emit and receive near-infrared light (at wavelengths between 650 and 950 nm), respectively. The emitted light passes through the scalp, tissue, and skull to reach the brain.^34^^–^^36^ The relationship between light attenuation (caused by absorption and scattering) and changes in the concentration of HbO and HbR can be expressed by the modified Beer–Lambert law (MBLL),^34^ since HbO and HbR have different absorption coefficients in the near-infrared wavelengths.^31^^,^^34^^,^^36^

Several recent studies have focused on building fNIRS-based BCI systems. In previous research, various types of experiments have been performed to measure the accuracy of systems’ classification, using mental arithmetic,^31^^,^^33^ motor imagery,^28^^–^^31^ motor execution,^23^^,^^30^^,^^33^^,^^37^ and other approaches. This type of research is particularly important since the final goal of the BCI is to have a system which is able to interpret subject intention, and any misclassification in the BCI system can lead to accidents for the user. Accordingly, improving classification accuracy is the most essential feature of the BCI-based communication system.^38^^,^^39^ To this end, it is important to exploit appropriate classifiers as well as discriminant features that can accurately represent the variability in the hemodynamic response signal.^40^

Many of the studies on fNIRS-based BCI primarily focused on different types of feature extraction techniques and machine learning algorithms.^40^ For feature extraction, methods of identifying statistical properties such as mean, slope, skewness, kurtosis, etc., from time-domain signals^40^ filter coefficients from continuous and discrete wavelet transforms (DWTs),^41^^,^^42^ and measurement based on joint mutual information^43^ have been used. In addition, for machine learning-based classification, methods such as linear discriminant analysis,^6^^,^^23^^,^^29^^,^^31^^,^^33^^,^^38^ support vector machine (SVM),^12^^,^^30^^,^^44^ hidden Markov model,^30^ and artificial neural network (ANN)^45^ have received considerable attention.

Among the above-mentioned techniques for feature extraction, most of the studies have relied on extracting the statistical values of the time-domain signal. However, reaching the highest classification accuracy depends on different factors, such as selecting the best set of combined features^46^ and the size of the time window.^31^ In addition, classification accuracies vary based on different mother wavelet functions for decomposition,^41^ which affect performance in a heuristic sense. To overcome the limitations of these conventional methods, therefore, an appropriate technique for feature extraction needs to be determined.

### 1.3 Objective

The results of previous studies have demonstrated that convolutional neural networks (CNNs) can successfully achieve high classification accuracy in many applications, including image recognition,^47^^,^^48^ artificial intelligence,^49^ speech detection, and multiple time-series processing.^50^^,^^51^ Considering CNNs’ ability to extract important features from a signal, CNN may be suitable for fNIRS-based BCI as well. Accordingly, our proposed method utilizes CNN to automatically extract the features from the hemodynamic response signal. To be specific, we attempt to answer the following two arguments: (1) does CNN outperform conventional methods in fNIRS-based BCI? (2) How well does CNN work with the input data of the hemodynamic response signal?

To address these questions, we compared the classification accuracies of CNN with those of conventional methods when used as the feature extractor and classifier in fNIRS-based BCI.^40^ Then, we analyzed how the trained convolutional filters in CNN optimized the features.

The rest of this work is organized as follows. In Sec. 2, the properties of the conventional methods as well as CNN are briefly introduced. Subsequently, data acquisition, preprocessing, and the proposed CNN structures are described in Sec. 3. Sections 4–6 cover the results, discussion, and conclusion, respectively.

## 2 Background

This section describes the details of the commonly used features, machine learning-based classifiers, and how the classification performance is evaluated for BCI systems.

### 2.1 Features Extracted from the Input Signal

While a large body of previous studies have reported various features which can be used to extract the hemodynamic signal, the most commonly used features for fNIRS-based BCI are signal mean ($\mu_{x_{i}}$), variance ($\sigma_{x_{i}}^{2}$), kurtosis ($K_{x_{i,j}}$), skewness ($S_{x_{i}}$), peak, and slope where such features are computed as^40^

$$\mu_{x_{i}}=\frac{1}{N}\overset{N}{\underset{j=1}{\sum }}x_{i,j},$$ (1)

$$\sigma_{x_{i}}^{2}=\frac{\overset{N}{\underset{j=1}{\sum }}{\left(x_{i,j}-\mu_{x_{i}}\right)}^{2}}{N},$$ (2)

$$K_{x_{i}}=\frac{\overset{N}{\underset{j=1}{\sum }}{\left(x_{i,j}-\mu_{x_{i}}\right)}^{4}/N}{\sigma_{x_{i}}^{4}},$$ (3)

and

$$S_{x_{i}}=\frac{\overset{N}{\underset{j=1}{\sum }}{\left(x_{i,j}-\mu_{x_{i}}\right)}^{3}/N}{\sigma_{x_{i}}^{3}},$$ (4)

where N is the total number of samples of $x_{i}$, $x_{i}$ is the i’th row of the input x, $x_{i,j}$ is the j’th signal amplitude of the input $x_{i}$, and $\sigma_{x_{i}}^{2}$ is the variance of $x_{i}$. The signal peak is computed by selecting the maximum value of $x_{i}$, and the slope is computed using linear regression.

### 2.2 Support Vector Machine

SVM is a discriminative classifier which optimizes a separating hyperplane by maximizing the distance between the training data.^52^ The decision boundary is obtained by

$$\min(\frac{1}{2}{\left\|w_{s}\right\|}^{2})+C\overset{L}{\underset{i=1}{\sum }}\epsilon^{\left(i\right)}\;s.t.\;y_{s}^{\left(i\right)}(x_{s}\cdot w_{s}+b_{s})-1+\epsilon^{\left(i\right)}\geq 0,$$ (5)

where $w_{s}$ is the weight vector, C>0 is the regularization parameter, $\epsilon^{i}>0$ is the training error, $y_{s}^{\left(i\right)}$ is the true class label for i’th input $x_{s}$, and $b_{s}$ is the bias. Among these, C plays an important role in reducing the error as well as accommodating the outliers through the training process of the data. In other words, it controls the trade-off between the data training error and the norm of the weights. As a matter of fact, determining a proper C is a vital step in training the SVM on the input data.^53^

### 2.3 Artificial Neural Network

**Fig. 1:**
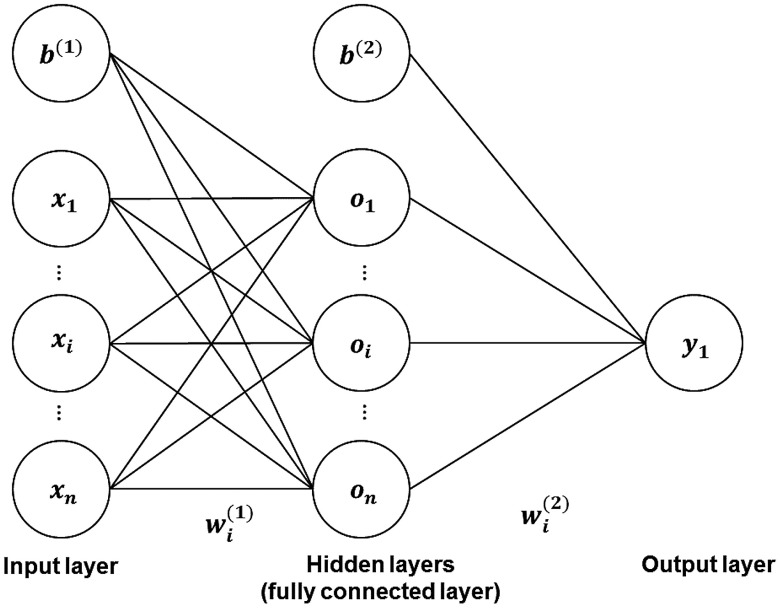
The common structure of ANN.

ANN is a classifier, inspired by a biological brain’s axon, with the ability to detect patterns in the training data set,^54^ which consists of assemblies of interconnected artificial neurons that provide nonlinear decision boundaries. Typically, ANN consists of multiple layers, respectively called the input layer, fully connected hidden layer(s), and the output layer, with one or more neurons in each layer (see Fig. 1). Through forward propagation, the output values are computed based on the activation function of the hidden layer(s) by

$$o_{i}=a[w^{\left(1\right)}\cdot x+b^{\left(1\right)}],$$ (6)

$$y_{i}=a[w^{\left(2\right)}\cdot o+b^{\left(2\right)}],$$ (7)

where $o_{i}$ is the output of the first fully connected hidden layer calculated by using an activation function a to transform the summation of bias value $b^{\left(1\right)}$, and the multiplication of the input vector x with the weight vector $w^{\left(1\right)}$. Likewise, $y_{i}$ is the output of the second fully connected hidden layer, which is similarly calculated by using the input vector o of the second layer, and the weight vector $w^{\left(2\right)}$ and the bias $b^{\left(2\right)}$.

Through the first iteration of the training procedure, weight values should be initialized. Proper weight initialization is one of the important operations for improving the classification performance of the networks.^55^ Afterward, the weight values are updated by the backward propagation by comparing the computed output values from the forward propagation with the desired output values, using a loss function. This iteration is performed until the minimum loss function value is achieved.^54^

To obtain a proper predictive model with ANN, several hyperparameters, such as learning rate, batch size, and number of epochs, should be considered. The learning rate is the parameter that controls how fast the weight values in the fully connected layers can be updated during the training process. Through the batch learning process, training data are separated into several sets, and this is followed by propagation through the training process, where the batch size is the number of samples in each set.^55^ An epoch is defined as the total number of times that the training procedure is completed.

### 2.4 Convolutional Neural Network

CNN is an effective classifier based on deep network learning. It is highly capable of automatically learning appropriate features from the input data by optimizing the weight parameters of each filter, using forward and backward propagation to minimize classification errors.^56^

**Fig. 2:**
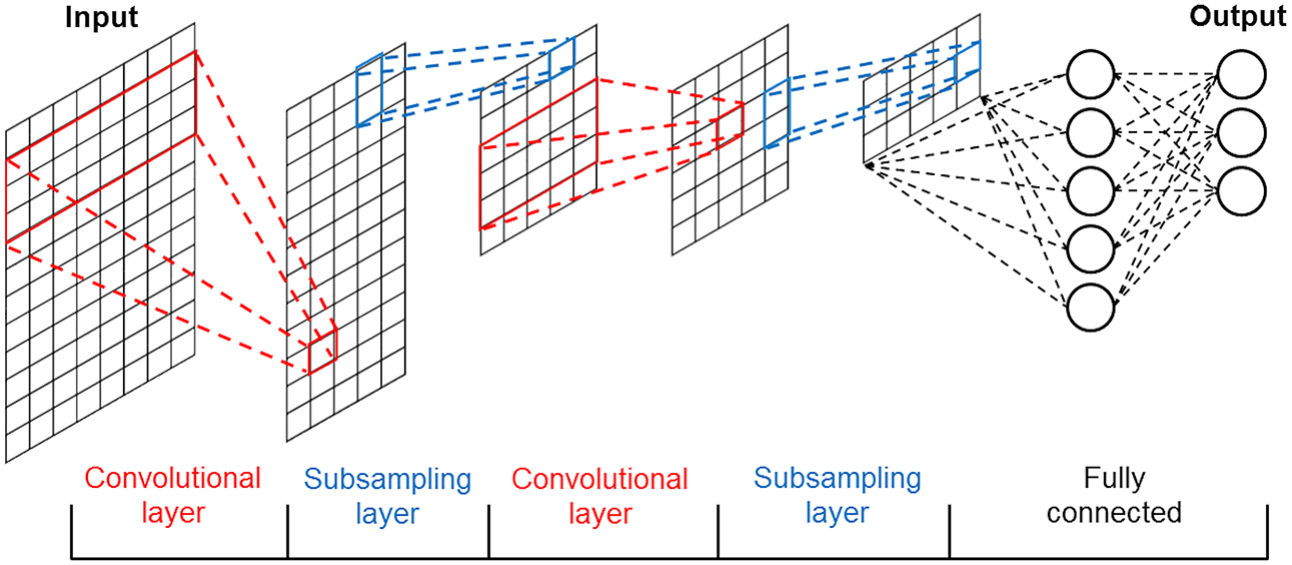
The common structure of convolutional neural network.

CNN consists of several layers, which are called the input layer, convolutional layer, fully connected hidden layer, and output layer (see Fig. 2). In the convolutional layers, a convolutional filter whose width is equal to the dimension of the input and kernel size (height) of h is convolved with the input data, where the output of the i’th filter is^57^

$$o_{i}=w\cdot x[i:i+h-1],$$ (8)

where w is the weight matrix, x[i:j] is the submatrix of input from row i to j, and $o_{i}$ is the result value.

Then, in order to build the feature map (the input of the next layer), the output of the convolutional layer is converted by an activation function similar to ANN. After each convolutional layer, additional subsampling operations such as max-pooling and dropout are performed to enhance the performance.

Max-pooling^57^ is one of the common methods used to reduce data size, and it stores only the important data. Dropout,^58^ which helps CNN avoid overfitting during the training process, is a regularization step that randomly drops out one or more hidden nodes. As with ANN, the mentioned hyperparameters such as learning rate, batch size, and number of epochs should be investigated for CNN in order to improve the classification performance.

### 2.5 Cross Validation

**Fig. 3:**
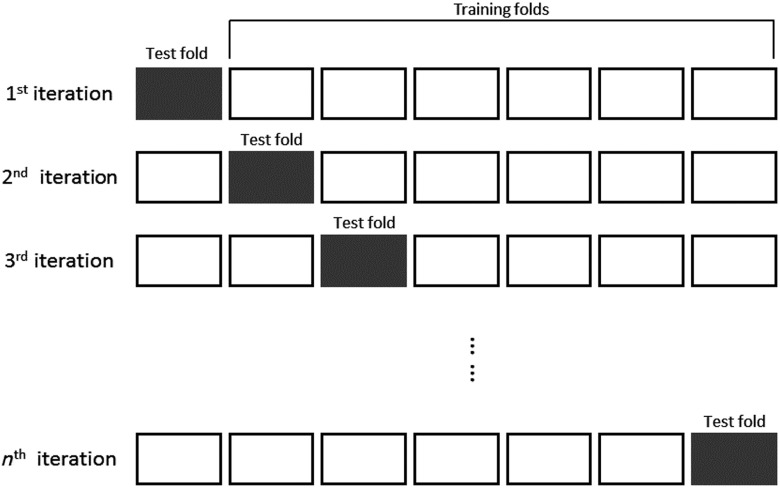
Cross-validation procedure.

k-fold cross validation is used to estimate the classification performance of the predictive model.^53^^,^^59^ The first step in this process is to divide the data into k-folds, where each fold contains an identical amount of the input data. Then, one fold is used as a test set, while the remaining folds are used as training sets (see Fig. 3). Afterward, a classification procedure is applied to the selected test and training sets. This process is performed for each of the k-folds, and the corresponding accuracies obtained from each test set are averaged to estimate the performance.

## 3 Method

### 3.1 Participants

Eight healthy subjects were recruited for the experiment (ages of 25.25±3.81 years, three females, all right-handed). The subjects were asked to avoid smoking and drinking alcohol or coffee within 3 h prior to the experiment. None of the subjects had been reported for any neurological or brain injuries. Written consent forms were obtained from all subjects. The experiment was approved by the Daegu Gyeongbuk Institute of Science and Technology (DGIST) Institutional Review Board (DGIST-170414-HR-004-01).

### 3.2 Data Acquisition

**Fig. 4:**
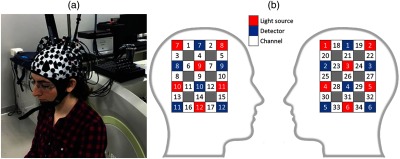
(a) A subject with optodes over motor area C3 and C4 based on the international 10–20 system and (b) the source and detector configuration. Channel numbers 1 to 17 and 18 to 34 were placed over motor areas C4 and C3, respectively.

For data acquisition, LABNIRS (Shimadzu), an fNIRS device with a multichannel continuous wave with three wavelengths (780, 805, and 830 nm) and a sampling rate of 25.7 Hz, was utilized. A total of 12 sources and 12 detectors, resulting in 34 measurement channels, were placed over the motor areas, C3 and C4, according to the international 10–20 system which corresponds to the motor cortex of the right- and left-hand motor execution (see Fig. 4).^60^ The distance between source and detector was 3 cm.

### 3.3 Experimental Procedure

The subjects sat on a comfortable chair in front of a computer screen, which displayed the experimental tasks. In the experiment, subjects were asked to perform a motor execution task in order to generate a robust signal for better discrimination. To be specific, while a black screen was displayed during the rest task, an arrow pointing right or left was shown during each of the right- or left-hand execution tasks, respectively.

**Fig. 5:**
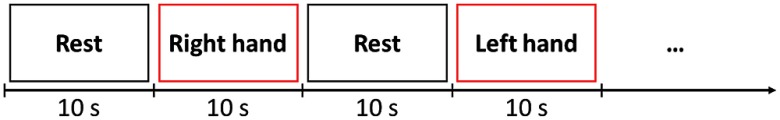
Experimental procedure includes rest and two motor tasks: right- and left-hand motor execution.

All of the subjects were asked to relax before the experiment in order to stabilize blood flow. For data acquisition, the subjects were trained to relax during the rest tasks and to perform finger tapping during the motor execution tasks. Each subject performed 10 experiments of five sessions of right- and left-hand motor executions, with two rest blocks per session (see Fig. 5). All the blocks lasted 10 s, and each block became a sample. The data for all the subjects were collected within three days. We eventually obtained a total of 100 samples of rest, 50 samples of right, and 50 samples of left-hand motor execution for each subject.

### 3.4 Acquired Data Preprocessing

#### 3.4.1 Calculation of hemoglobin concentration changes

After signal measurement, we converted the signals of light intensity into concentration changes of HbO and HbR by MBLL, utilizing statistical toolbox NIRS-SPM.^61^ The MBLL equation is given by

$$[\begin{array}{c} \Delta [\mathrm{HbO}]\\ \Delta [\mathrm{HbR}] \end{array}]=\frac{1}{d\cdot \mathrm{DPF}}{\left[\begin{array}{cc} \epsilon_{\lambda 1}^{\mathrm{HbO}} & \epsilon_{\lambda 1}^{\mathrm{HbR}}\\ \epsilon_{\lambda 2}^{\mathrm{HbO}} & \epsilon_{\lambda 2}^{\mathrm{HbR}} \end{array}\right]}^{-1}[\begin{array}{c} \Delta {\mathrm{OD}}_{\lambda 1}\\ \Delta {\mathrm{OD}}_{\lambda 2} \end{array}],$$ (9)

where Δ[HbO] and Δ[HbR] are the changes in HbO and HbR concentration, respectively, d is the distance between the light source and detector, DPF is the differential path length factor, ϵ is the extinction coefficient at wavelength λ, and ΔOD is the optical density change.

#### 3.4.2 Filtering

The acquired hemodynamic signal contains various physiological noises, including the heart rate at 0.8 Hz, respiration at 0.2 Hz, Mayer wave at 0.1 Hz, and very-low-frequency oscillations at 0.03 Hz.^36^^,^^38^^,^^40^ Among various possible criteria, we employed wavelet filtering to remove physiological noise.^62^

The wavelet transform is an efficient method of signal analysis and performs by adjusting its window width in both time and frequency domains. For denoising a signal S[n], first, wavelet coefficients are obtained by shifting and dilating the waveforms of the so-called mother function ψ[n], and then important coefficients are selected to reconstruct the signal by thresholding. For a more comprehensive analysis, we also exploited multiresolution analysis (MRA) which decomposes signals into a tree structure using the DWT.^41^^,^^63^ Using MRA based on DWT, S[n] can be approximated by expanding both low- and high-frequency coefficients for M time points as

$$S[n]=\frac{1}{\sqrt{M}}\underset{k}{\sum }A_{\phi }[j_{0},k]\phi_{j_{0},k}[n]+\frac{1}{\sqrt{M}}\overset{\infty }{\underset{j=j_{0}}{\sum }}\underset{k}{\sum }D_{\psi }[j,k]\Psi_{j,k}[n],$$ (10)

where $\phi_{j_{0},k}[n]$ and $\Psi_{j,k}[n]=\frac{1}{\sqrt{j}}\psi (\frac{n-k}{j})$ are scaling and wavelet mother functions, respectively, in which the mother function ψ[n] is dilated with scaling parameter j, translated by k which is the number of decomposition levels, and represented as $\Psi_{j,k}[n]$. Since these functions are orthogonal to each other, taking the inner product results in obtaining the approximation coefficients (low frequency) $A_{\phi }[j_{0},k]=\frac{1}{\sqrt{M}}\underset{n}{\sum }S[n]\phi_{j_{0},k}[n]$ and the detailed coefficients (high frequency) $D_{\psi }[j,k]=\frac{1}{\sqrt{M}}\underset{n}{\sum }S[n]\Psi_{j,k}[n]$. By denoting

$$a_{j_{0}}=\frac{1}{\sqrt{M}}\underset{k}{\sum }A_{\phi }[j_{0},k]\phi_{j_{0},k}[n],$$ (11)

and

$$d_{j}=\frac{1}{\sqrt{M}}\underset{k}{\sum }D_{\psi }[j,k]\Psi_{j,k}[n],$$ (12)

Eq. (10) can be rewritten as

$$S[n]=a_{j_{0}}+\underset{j}{\sum }d_{j}.$$ (13)

In order to remove the undesired high- and low-frequency noises, we exploited a 10-level wavelet decomposition with a Daubechies (db5) mother function.^62^ In addition, we used a bandpass frequency between 0.02 and 0.1 Hz, in which the combination of low-frequency components $d_{8}$ and $d_{9}$ from the 10-level decompositions was solely in the same 0.02- to 0.1-Hz frequency range. Therefore, the filtered signal was reconstructed based on $d_{8}$ and $d_{9}$ by $S_{\text{denoised}}[n]=d_{8}+d_{9}$. After filtering, the hemodynamic response signals were normalized into range (0,1) by subtracting with the signal mean and scaling.

### 3.5 Feature Extraction and Classification

After filtering, we trained and tested the classifiers for each individual subject based on the extracted features. Following the training step, we computed the classification accuracies from both the conventional methods (SVM- and ANN-based fNIRS) and the proposed method (CNN-based fNIRS). In this section, we discuss the details of the conventional methods and our proposed CNN structure.

#### 3.5.1 Conventional methods

As mentioned, features were extracted after the filtering step, followed by normalizing into range (0,1). The obtained input data contained 408 feature dimensions (6 features ×2 signal of HbO and HbR ×34 channels). Using such features with the settings above, we evaluated the performance of the conventional methods by observing the concentration changes of HbO and HbR over all channels using SVM and ANN.

Before applying SVM, since such high-dimensional features usually suffer from performance degradation in classifiers,^64^ a principle component analysis (PCA) was utilized to decrease the dimensions of the data. This reduces the aforementioned effect by maximizing the variance using a smaller number of principle components.^52^ Grid search^53^^,^^65^ was used to determine the number of principle components and the C regularization parameters in SVM, and the combination of both parameters which yielded the highest classification accuracy was selected.

In this study, we report the results for linear SVM and multiple structures of ANN (see Table 1). To be specific, structures of ANN with one hidden layer (ANN1) and two hidden layers (ANN2) were evaluated. For further comprehensive investigation, each structure of ANN was considered with various numbers of neurons. All of the aforementioned hyperparameters were tuned for each subject (see Table 2).

**Table 1:** Structures of ANN.

Structure	Hidden layer	Neurons in each hidden layer
ANN1-a	1	128
ANN1-b	1	256
ANN1-c	1	512
ANN2-a	2	256, 128
ANN2-b	2	512, 256
ANN2-c	2	512, 128

**Table 2:** Hyperparameters of each individual subject for ANN.

Subject	Parameters	ANN1-a	ANN1-b	ANN1-c	ANN2-a	ANN2-b	ANN2-c
1	Epochs	50	50	100	20	100	100
	Batch size	64	64	16	16	16	32
	Learning rate	0.001	0.0005	0.001	0.0005	0.001	0.001
2	Epochs	100	100	100	100	50	50
	Batch size	16	16	32	16	16	16
	Learning rate	0.0005	0.001	0.0005	0.0001	0.0005	0.001
3	Epochs	100	100	50	50	50	100
	Batch size	16	16	64	64	32	32
	Learning rate	0.0001	0.0001	0.0005	0.0001	0.0001	0.0001
4	Epochs	50	100	50	50	100	100
	Batch size	16	32	64	32	32	64
	Learning rate	0.0005	0.0005	0.0005	0.0005	0.0001	0.0005
5	Epochs	100	100	100	100	100	100
	Batch size	32	64	64	16	64	64
	Learning rate	0.0005	0.0005	0.0005	0.0001	0.001	0.001
6	Epochs	100	50	100	100	100	100
	Batch size	16	16	32	16	16	16
	Learning rate	0.001	0.001	0.0005	0.0005	0.0005	0.0005
7	Epochs	100	100	100	100	50	100
	Batch size	16	32	16	16	16	64
	Learning rate	0.0005	0.0005	0.001	0.0005	0.0005	0.001
8	Epochs	100	100	50	50	50	100
	Batch size	64	64	32	32	32	16
	Learning rate	0.0001	0.0001	0.0001	0.0001	0.0001	0.0001

#### 3.5.2 Proposed structures of convolutional neural network

Instead of using other methods, we employed CNN as the feature extractor as well as the classifier in this study. As the input data, the changes in HbO and HbR concentration over all channels were passed through CNN layers using the structures presented in Table 3. The input data for CNN were an M by N matrix, where M is the number of points during 10 s that correspond to the sampling rate (M=time×sampling rate≈257) and N is the number of channels for both HbO and HbR (34 channels each of HbO and HbR). Similar to the process used to evaluate the conventional methods, we considered two structures of CNN, that is, CNN with one convolutional layer (CNN1) and three convolutional layers (CNN2). Furthermore, each structure of CNN was considered with a distinct number of filters (see Table 3).

**Table 3:** Structures of CNN.

Structure	Convolutional layer	Filters in each convolutional layer
CNN1-a	1	32
CNN1-b	1	64
CNN2-a	3	32, 32, 32
CNN2-b	3	64, 64, 64

**Fig. 6:**
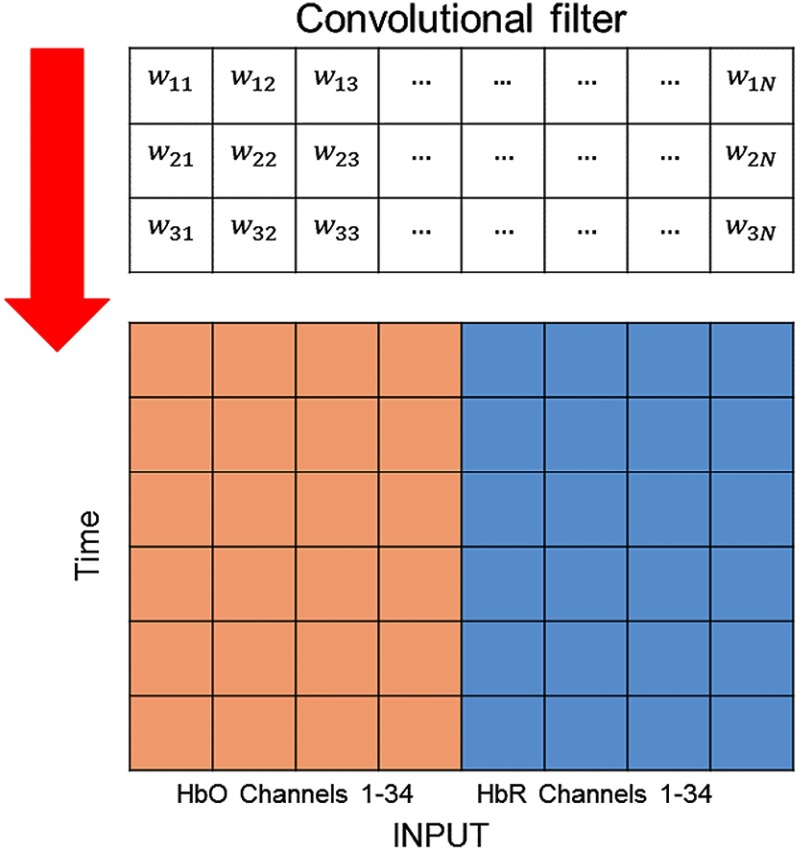
The input data consisted of the concentration changes of HbO (red) and HbR (blue) overall channels. A convolutional filter ran through the input data along the vertical axis.

All of the convolutional filters in the convolutional layers performed one-dimensional convolution with the input data along the vertical axis, as shown in Fig. 6. Each convolutional layer consisted of filters with a kernel size of 3, and an algorithm^63^ was used to update the weight values in the training process. After each convolutional layer, max-pooling with a kernel size of 2 was applied, followed by dropout with a dropout rate of 50%. The first and second fully connected layers contained 256 and 128 hidden nodes, respectively. The output layer had 3 nodes corresponding to the three classes, which were classified using softmax. For better understanding of the structures mentioned here, the input and output sizes of each layer in our proposed CNN2-a are summarized in Table 4.

**Table 4:** Input and output size of the CNN2-a.

Layer	Input size	Output size	Properties
Convolutional layer 1	257, 68	257, 32	32 filters with kernel size 3
Max-pooling 1	257, 32	128, 32	Kernel size 2
Dropout 1	128, 32	128, 32	Dropout rate 50%
Convolutional layer 2	128, 32	128, 32	32 filters with kernel size 3
Max-pooling 2	128, 32	64, 32	Kernel size 2
Dropout 2	64, 32	64, 32	Dropout rate 50%
Convolutional layer 3	64, 32	64, 32	32 filters with kernel size 3
Max-pooling 3	64, 32	32, 32	Kernel size 2
Dropout 3	32, 32	32, 32	Dropout rate 50%
Fully connected layer 1	1024	256	256 hidden nodes
Fully connected layer 2	256	128	128 hidden nodes
Output layer	128	3	3 hidden nodes

In the proposed structure, the activation functions of all layers were set to a rectified linear unit (ReLU), which is a nonlinear function, as shown in^66^

$$a(x)=\{\begin{array}{l} 0,x<0\\ x,x\geq 0 \end{array}.$$ (14)

Unlike other activation functions, ReLU avoids a vanishing gradient and in practice converges to the optimum point much faster. Consequently, it improves the training process of deep neural network architectures on large scale and complex data sets.

In addition, the hyperparameters for training all the CNN structures, including learning rate, number of epochs, and batch size, were chosen for each individual subject using Grid search (see Table 5). Adam was applied as a gradient descent optimization algorithm, whose parameters $\beta_{1}$, $\beta_{2}$, and ε were set to 0.9, 0.1, and $10^{-8}$, respectively.^67^

**Table 5:** Hyperparameters of each individual subject for CNN.

Subject	Parameters	CNN1-a	CNN1-b	CNN1-c	CNN2-a
1	Epochs	100	100	100	100
	Batch size	16	64	32	16
	Learning rate	0.001	0.001	0.001	0.0005
2	Epochs	50	50	100	100
	Batch size	32	16	16	64
	Learning rate	0.0005	0.001	0.0005	0.001
3	Epochs	50	100	50	100
	Batch size	16	64	64	32
	Learning rate	0.0001	0.0005	0.001	0.0005
4	Epochs	100	100	100	100
	Batch size	32	32	16	16
	Learning rate	0.0001	0.001	0.0001	0.0005
5	Epochs	50	50	100	100
	Batch size	64	16	32	64
	Learning rate	0.001	0.0005	0.001	0.001
6	Epochs	100	100	50	100
	Batch size	16	32	16	32
	Learning rate	0.0005	0.0001	0.001	0.001
7	Epochs	50	100	100	100
	Batch size	64	32	64	16
	Learning rate	0.001	0.001	0.0005	0.0005
8	Epochs	50	100	100	100
	Batch size	64	32	64	16
	Learning rate	0.001	0.001	0.0005	0.0005

### 3.6 Visualization of Feature Extraction

Many previous studies of feature extraction in fNIRS-based BCI have been reported in the past. Since appropriate features and classifiers are desired in order to achieve high classification accuracy, the proposed method of the CNN exploitation was utilized in this study because of its automatic feature extraction property.

To provide better insights into the feature extraction performance, a visualization of the features extracted by the aforementioned methods is shown and compared. Because high-dimensional data are difficult to visualize, the PCA was applied to reduce the dimensionality of the data.

**Fig. 7:**
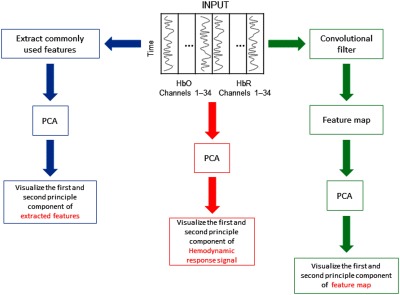
The overall procedure to visualize signal features, including the hemodynamic response signal, commonly used features in fNIRS-based BCI, and output of the convolutional filter (feature map). The first and second principle components of the signal features are illustrated for the visualization.

In this study, we also compared the visualization of the hemodynamic response signals with the features extracted by conventional methods and convolutional filter, by plotting the first two principle components of the PCA. The overall procedure to visualize signal features is shown in Fig. 7.

### 3.7 Computational Time in the Classification

In our work, various machine learning algorithms were applied to classify tasks, including rest, right-, and left-hand motor executions. For the ANN and CNN, we trained the model using GPU GeForce GTX 1070.^68^ The data were divided equally into 10-folds, and then nine folds were used as a training set. To imitate the environment of a real application, a single sample was fed through the trained model then the computational time was measured. For training SVM, ANN, and CNN, the hyperparameters such as C regularization, number of epochs, and learning rate were set to 1, 1, and 0.01, respectively.

## 4 Results

### 4.1 Measured Hemodynamic Responses

**Fig. 8:**
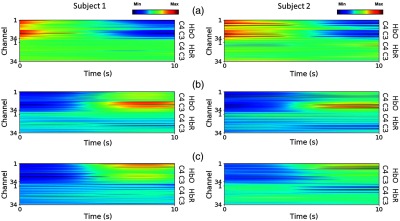
Average hemodynamic response of each execution task measured from subject 1 and 2: (a) rest, (b) right-, and (c) left-hand motor execution. Each input presents concentration changes of HbO and HbR overall 34 channels. Red and blue colors represent the maximum and minimum amplitude, respectively.

In the experiment, the changes in HbO and HbR concentration were measured as the input data for classification. The average of the hemodynamic response signals was obtained with respect to the samples from subjects 1 and 2, across full sessions of each task for rest, right-, and left-hand motor executions and are shown in Figs. 8(a)–8(c), respectively. Each row of the input data indicates signal amplitudes. These are represented by red and blue colors, which imply the maximum and minimum amplitudes, respectively. The beginning and the end of the tasks correspond to 0 and 10 s, respectively.

As is widely known, neural activity induces typical changes in cerebral blood oxygenation, resulting in increases in HbO concentration and decreases in HbR concentration.^34^ In our results, a similar behavior in the hemodynamic response can be observed, as shown in Fig. 8. To be specific, the signals obtained from channels over C3 show higher cortical activation of HbO over a period of 5 to 10 s during the right-hand motor execution [see Fig. 8(b)], whereas the signals over C4 have higher activation during the left-hand motor execution [see Fig. 8(c)].

**Fig. 9:**
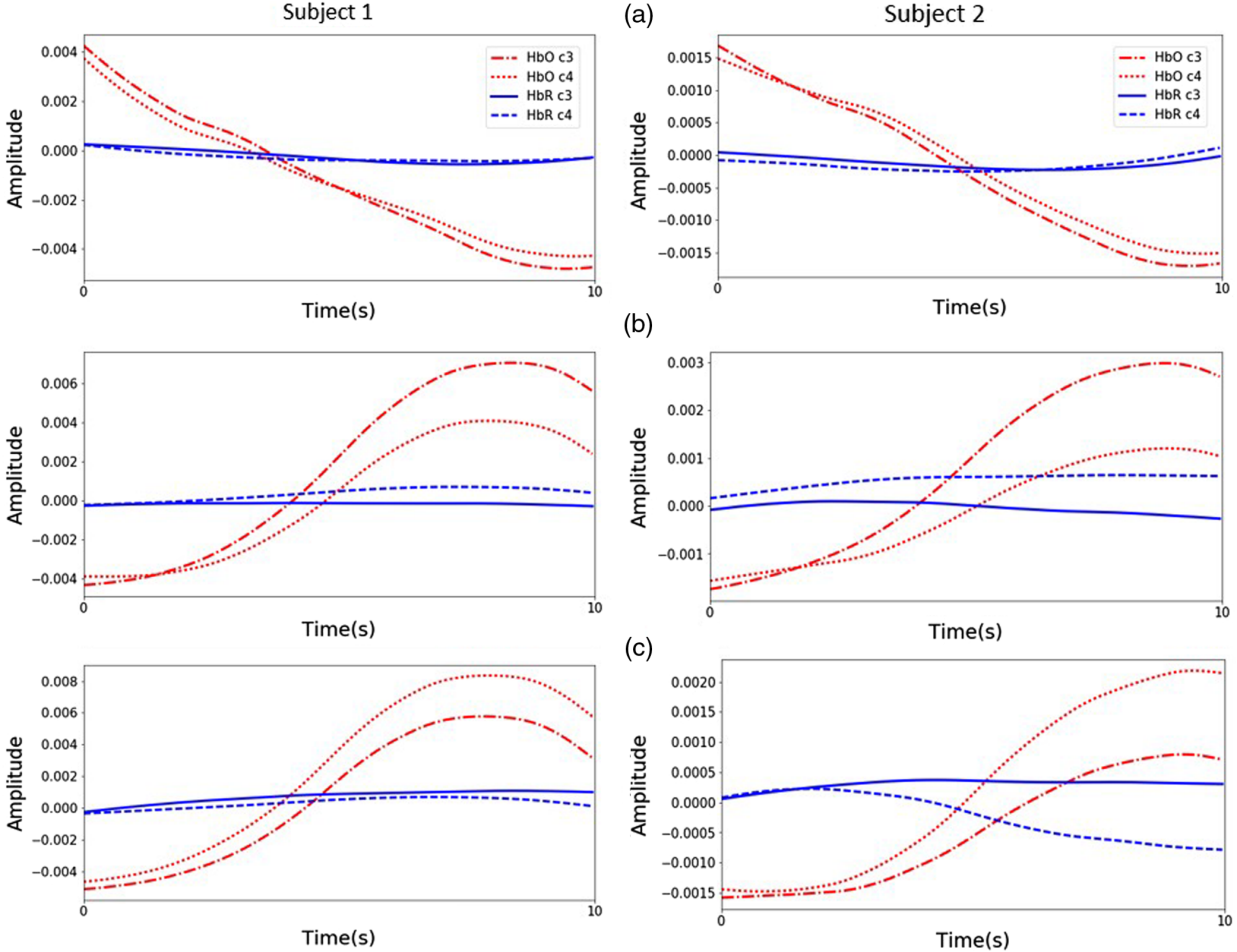
Average signal amplitude of subjects 1 and 2 across left (C3) and right (C4) hemisphere from full sessions of each class: (a) rest, (b) right-, and (c) left-hand motor execution. Red and blue colors imply HbO and HbR, respectively. Solid and dot lines are related to the C3 and C4 motor areas in that order.

Figure 9 shows the averaged signals for the entire experiment over all channels of the left and right hemispheres. It is obvious that the change in HbO concentration is higher in the left cerebral cortex during the right-hand motor execution [see Fig. 9(b)], while it is larger in the right cerebral cortex during the left-hand motor execution [see Fig. 9(c)]. The brain behaviors observed in Fig. 9 demonstrate that three-class discrimination, for rest, right-, and left-hand motor execution, can be achieved, since they show different patterns of cortical activation over the left and right hemispheres.

### 4.2 Classification Accuracies

To determine the classification accuracies of the SVM, ANNs, and CNNs, we employed 10-fold cross validation to estimate performance and to optimize hyperparameters, as we attempted to discriminate the three classes of rest, right-, and left-hand motor execution. In this section, the classification accuracies of commonly used features classified by SVM and ANNs are compared with those obtained by CNNs.

**Fig. 10:**
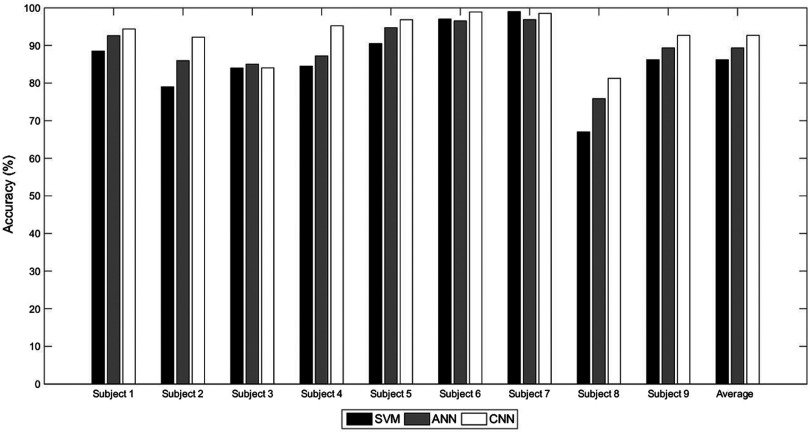
Average classification accuracies of the individual subjects.

To be specific, the classification accuracies for all the tested criteria for the individual subjects are presented in Table 6. As expected, the results of all the individual subjects indicate that the use of CNN was significantly superior to SVM and ANN. For convenience of analysis, the average of the classification accuracies of SVM, ANN, and CNN (86.19%, 89.35%, and 92.68%, respectively) are presented in Fig. 10, which confirms the superior performance of CNN over the conventional methods. This superior performance is due to CNN’s ability to learn the inherent patterns of the input data, by updating the weight values of the convolutional filters.

**Table 6:** Classification accuracies of the individual subjects (%).

	S1	S2	S3	S4	S5	S6	S7	S8	Average
SVM	88.50	79.00	84.00	84.50	90.50	97.00	99.00	67.00	86.19
ANN1-a	91.67	85.33	84.83	85.00	94.20	96.17	96.50	76.33	88.75
ANN1-b	92.83	83.83	84.67	87.67	94.30	96.00	96.33	75.00	88.83
ANN1-c	92.83	85.67	85.17	87.50	94.50	96.50	96.67	75.83	89.33
ANN2-a	93.67	86.67	84.33	87.50	95.67	96.50	96.83	75.67	89.61
ANN2-b	92.50	86.17	85.67	88.67	94.83	97.00	97.17	76.08	89.76
ANN2-c	92.17	88.00	85.50	87.00	94.83	97.00	97.67	76.17	89.79
CNN1-a	95.00	91.67	84.83	95.67	97.33	99.00	98.67	80.33	92.81
CNN1-b	95.33	92.17	85.83	96.00	96.83	99.00	99.00	80.50	93.08
CNN2-a	94.33	91.83	82.17	95.00	96.67	98.67	98.33	82.17	92.40
CNN2-b	92.83	93.17	83.33	94.33	96.50	99.00	98.17	82.00	92.42

The learning performance can be affected by the size of the training set, and this is especially true for ANN and CNN, where a larger-sized training set usually provides higher classification performance. To examine the effect of the size of the data set on the classification accuracy, the average classification accuracies across all the subjects were obtained, based on different numbers of samples.

**Fig. 11:**
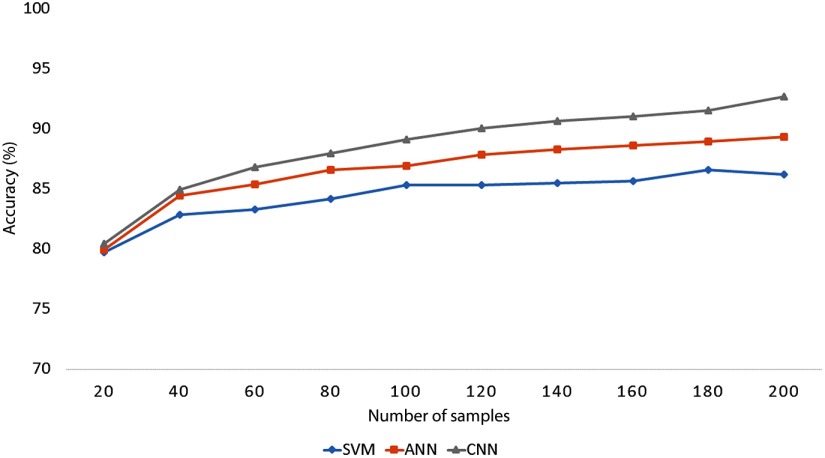
Average classification accuracies across all the subjects, based on different number of samples.

To evaluate the classification performance, 10-fold cross validation was utilized. For all the classification methods, the classification performance was found to increase with the number of samples in the data set, and the classification accuracy of CNN outperformed other tested methods for all numbers of samples (see Fig. 11). Moreover, the CNN was also able to attain higher accuracy with smaller numbers of samples; for instance, CNN exceeded 90% accuracy with 120 samples, whereas ANN required 200 samples to reach 89% accuracy.

### 4.3 Analysis of Feature Extraction Performance

**Fig. 12:**
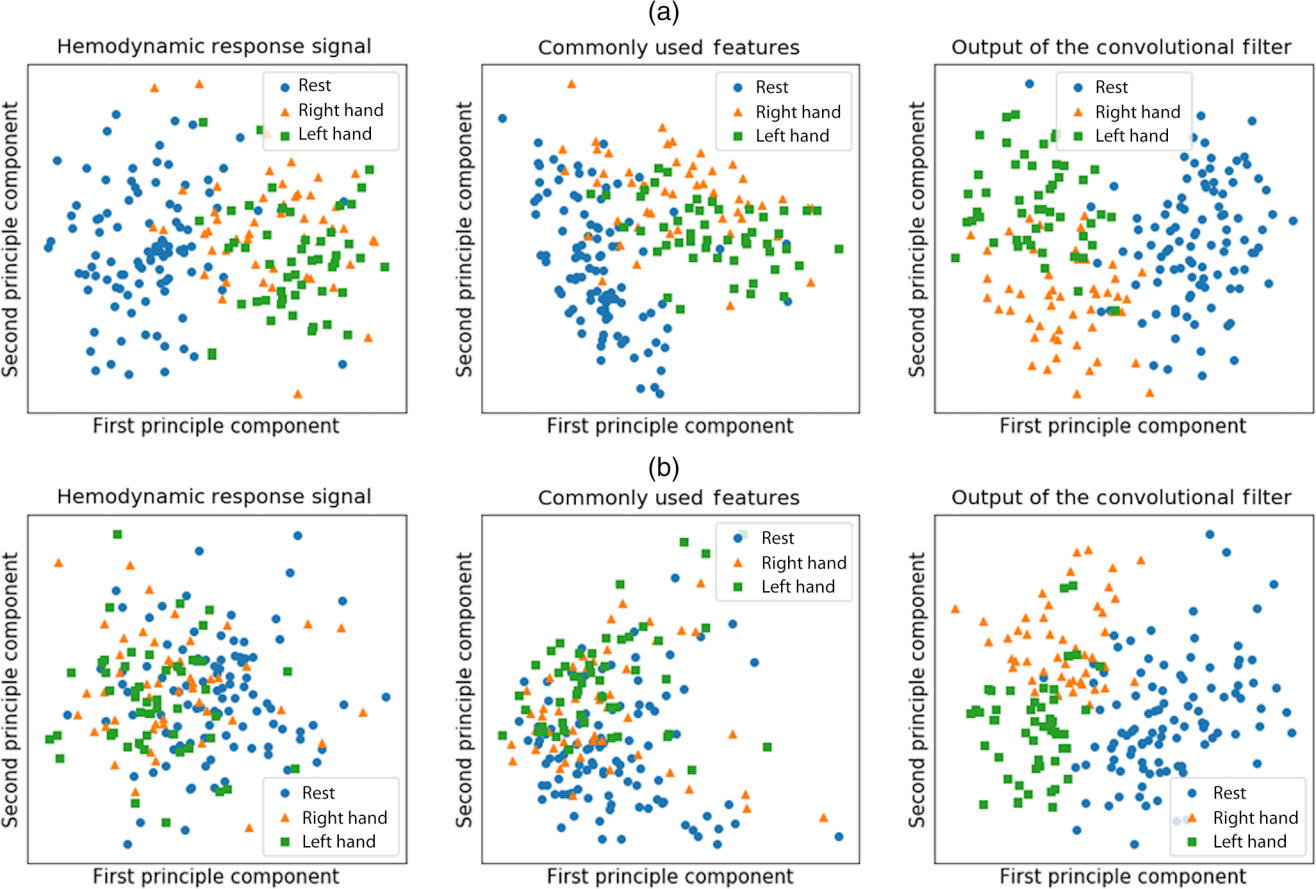
The visualization of the hemodynamic response signals, commonly used features, and output of the convolutional filter from (a) subject 1 and (b) subject 2.

To better understand the feature extraction performance, we visualized the three classes of rest, right-, and left-hand motor executions. To be specific, three classes were visualized using the hemodynamic response signals, features extracted by the conventional methods, and the output of the first layer convolutional filter, by plotting the first and second principle components of PCA (see Fig. 12). The results for subjects 1 and 2 show that the features extracted by the convolutional filters are better discriminated compared with commonly used features and the hemodynamic response signals.

When considering just the binary classification of rest and motor execution, both the conventional methods and CNN resulted in well-separable features. However, for the binary classification of right- and left-hand motor executions, and for multiclass classification, it was clear that features extracted by the convolutional filter were better discriminated as compared with the conventional methods.

### 4.4 Convolutional Filters of Convolutional Neural Network

One might notice that CNN is able to recognize the patterns of three different classes by updating its filters’ weight values. Therefore, to further investigate the convolutional filters of CNN, we examined the first layer of CNN to determine whether it is able to identify the distinguishable channels from the input or not. By training the data using forward and backward propagations, we let CNN learn how to emphasize some channels containing distinguishable signals by increasing the corresponding weight values, since each column of convolutional filter interacts with each channel from the input data. To approximate the most distinguishable channel, each column of the convolutional filter was averaged after training. Then, the channel of all of the samples of the input data with the highest weight value of the averaged convolutional filter was selected for visualization.

**Fig. 13:**
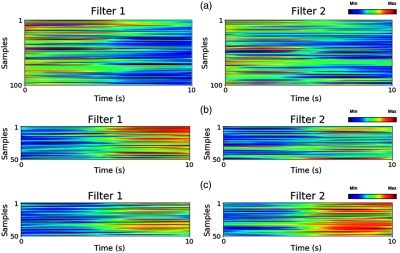
Each filter trained by subject 1 represents signals from a channel in every samples corresponding to the highest weight value. The filters represent three classes in the classification: (a) rest, (b) right-, and (c) left-hand motor execution.

In order to visualize the essential information, the most distinguishable channels from all the samples were selected. Two examples of the CNN filter weight values from subject 1 are shown in Fig. 13, where each row represents the most distinguishable signal from a single sample and the red and blue colors indicate the maximum and minimum amplitudes, respectively. We found that over a period of 5 to 10 s, there were remarkable differences in the signals chosen from both filters for the three classes of rest, right-, and left-hand motor execution.

Subsequently, in Fig. 13(a) which represents the rest task, both filters have low signal amplitude. Figure 13(b) represents the right-hand motor execution, in which filter 1 shows a higher signal amplitude than filter 2. In the same manner, Fig. 13(c) shows the left-hand motor execution, in which filter 2 exhibits a higher signal amplitude compared with filter 1. Therefore, it can be concluded that filter 1 can detect right-hand motor execution, and filter 2 detects left-hand motor execution.

### 4.5 Computational Time

The computational time for each of the classification algorithms, i.e., SVM, ANN, and CNN, was averaged across all subjects and structures (see Table 7). For the training process, the computational time for CNN was ∼2 and 183 times greater than ANN and SVM, respectively. For testing time, the computational time for CNN was ∼6 and 81 times greater than ANN and SVM, respectively. The computational time for CNN in the training and testing process was longer than ANN and SVM, as its structure is deeper and more complex. However, it provides a better performance in terms of classification accuracy.

**Table 7:** Computational time(s).

	Training time	Testing time
SVM	0.00645	0.00059
ANN	0.63299	0.00734
CNN	1.17945	0.04751

## 5 Discussion

The primary aim of the present study was to evaluate the use of CNN versus conventional methods in fNIRS-based BCI, particularly in light of the automatic feature extraction property of CNN. The proposed and conventional methods were investigated to compare their respective classification accuracies.

In the experiment, motor execution tasks performed by healthy subjects were utilized to obtain strong and robust hemodynamic response signals. However, in real applications, motor imagery can produce a greater impact than motor execution tasks, in both healthy users and in patients with severe motor impairment. A previous study reported that the cortical activation resulting from motor execution is similar to motor imagery.^6^ Hence, it is feasible that a healthy user or a patient without a brain injury, such as SCI, will be able to use motor imagery for commands instead of motor execution. Further investigation of the use of motor imagery, and the study of patients with neurological disorders, will be explored in the future.

The results of the classification accuracies in Fig. 10 imply that the proposed method using CNN outperforms the conventional methods. To be specific, the analysis of signal features by visualizing the first and second principle components demonstrates that the features extracted by the convolutional filter yield better discriminating features than conventional methods, because it is capable of learning appropriate features from the training data.

Additionally, the channels corresponding to the highest weight value in the trained CNN filter demonstrate that the convolutional filter emphasizes the discriminating signal from the training data. It is also worthwhile to note that while the performance of feature extraction for the binary classification of rest and motor execution was similar for both the conventional and proposed methods, since they showed well-discriminated features, the proposed method performed better for multiclass data. This is because the convolutional filter is able to transform mixed data into well-separated data.

Consequently, the proposed method will be appropriate for various systems that require multitasks to command. For instance, a brain-controlled wheelchair requires multiclass classification to control the wheelchair in several directions. Although the proposed method requires a longer time for training, it performs better in multiclass classification.

The number of samples used to train the classifier directly affects classification accuracy, especially in the complex classifier, as shown in Fig. 11. For a small number of samples, the classification performances of SVM, ANN, and CNN were similar. However, as the number of samples increased, the complex classifier was able to achieve higher accuracy than the simple classifier, though the computational time to train was much greater than that of the simple classifier.

This means there is a trade-off between accuracy and ease of use when building the appropriate BCI system. The user must take a longer time to train the complex classifier to obtain a high-performance classifier. When an application requires ease of use over safety, the conventional methods might be more appropriate, since a shorter time and smaller-sized training set are desired.

On the other hand, in the case of vital applications, systems to control assistive technology devices for a patient with motor impairment require very high accuracy, since any misclassification would probably lead to a serious accident. Consequently, in such cases the proposed method is recommended even if it takes a longer time, because it achieves higher accuracy with a smaller number of samples (see Fig. 11).

## 6 Conclusions

To enhance the classification accuracy of an fNIRS-based BCI system, we applied CNN for automatic feature extraction and classification, and compared those results with results from conventional methods employing SVM and ANN, with features of mean, peak, slope, variance, kurtosis, and skewness. From the measurement results for rest, right-, and left-hand motor execution on eight subjects, the CNN-based scheme provided up to 6.49% higher accuracy over conventional feature extraction and classification methods, because the convolutional filters can automatically extract appropriate features.

The results confirmed that there was an improvement in accuracy when using CNN over the conventional methods, which can lead to the practical development of a BCI system.

Since classification accuracy is the most essential factor for many BCI applications, we will explore further improvements in the accuracy of fNIRS-based BCI by implementing various deep learning techniques, as well as combining fNIRS with other neuroimaging modalities. To investigate clinical applications, we will also undertake experiments with patients.
